# Comparative Analysis of Fecal Microbiota and Metabolomic Profiles in Male and Female Leizhou Goats Offered a 10% Crude Protein Diet Among Four Energy Levels

**DOI:** 10.3390/ani15152174

**Published:** 2025-07-23

**Authors:** Hu Liu, Wenji Wang, Weishi Peng, Anmiao Chen, Xiaogao Diao, Xia Yang, Jianmin Chai, Yuanting Yang, Ke Wang, Jiancheng Han, Hanlin Zhou

**Affiliations:** 1 Zhanjiang Experimental Station, Chinese Academy of Tropical Agricultural Sciences, Zhanjiang 524013, China; m17378095519@163.com (W.P.); cam1835287831@163.com (A.C.); ytyang10@163.com (Y.Y.); lp_wangke@163.com (K.W.); hanjiancheng810@163.com (J.H.); 2 Department of Animal and Veterinary Sciences, Aarhus University, DK 8830 Tjele, Denmark; wangwj@anivet.au.dk; 3 Sanya Institute, China Agricultural University, Sanya 572024, China; b20193040335@cau.edu.cn; 4 Environment and Plant Protection Institute, Chinese Academy of Tropical Agricultural Sciences, Haikou 571101, China; yangx2025@catas.cn; 5 Guangdong Provincial Key Laboratory of Animal Molecular Design and Precise Breeding, School of Animal Science and Technology, Foshan University, Foshan 528225, China; jchai@fosu.edu.cn

**Keywords:** goats, gender, fecal microbiome, low-protein diet differ with energy levels

## Abstract

The gut microbiota plays a crucial role in maintaining the host’s metabolism and can influence the host’s productivity. Both dietary composition and gender have distinct effects on the composition of the gut microbiota. Therefore, to investigate the differences in the structure and function of the gut microbiota between female and male goats, we analyzed their fecal microbiota and metabolites when fed a 10% crude protein diet at four different energy levels—7.01, 8.33, 9.66, and 10.98 MJ/kg DM. Four non-pregnant female and four male Leizhou goats (all 8 months of age) were used in the experiment, with an average body weight of 10.3 ± 0.8 kg for females and 13.6 ± 1.1 kg for males (mean ± SD). The animals were assigned to two separate 4 × 4 Latin square designs according to their gender, each consisting of four treatments and four 28-day periods, including 25 days of a dietary adaptation period and 3 days of fecal sample collection per period. The data were analyzed using the SAS statistical package and Pearson’s correlation analysis. The dominant phyla for all samples were Firmicutes and Bacteroidota, regardless of dietary energy levels or gender. Among fecal bacteria, *unclassified_f_Lachnospiraceae* was the dominant genus in the female goats, and *Oscillospiraceae_UCG-005* was the dominant genus in the male goats. The relative abundance of *unclassified_f_Lachnospiraceae* (*p* < 0.001), *Bacteroides* (*p* = 0.007), *norank_f_Ruminococcaceae* (*p* = 0.024), *Mediterraneibacter* (*p* = 0.001), and *norank_f_Muribaculaceae* (*p* = 0.008) was greater in the female goats than in the male goats. In contrast, the relative abundance of *Oscillospiraceae*_UCG-005 (*p* < 0.001), *Ruminococcus* (*p* = 0.035), *Monoglobus* (*p* = 0.006), *Oscillospiraceae-NK4A214_group* (*p* = 0.008), *norank_f_F082* (*p* < 0.001), and *Prevotellaceae_UCG-003* (*p* < 0.001) was lower in the female goats than in the male goats. The volcano plot showed that there were 153, 171, 171, and 183 differential metabolites between the female and male goats at dietary energy levels of 7.01, 8.33, 9.66, and 10.98 MJ/kg DM, respectively. Numerous correlations were observed between differential metabolites and microflora genera. We concluded that the non-pregnant female and male goats exhibited distinct metabolic abilities when consuming a 10% crude protein diet at four different energy levels. Interestingly, in the female and male goats, the fecal microbiota also showed some differing responses to the energy levels. These results provide a gender-based reference for formulating low-protein dietary strategies for 8-month-old Leizhou goats.

## Simple Summary

Energy and protein are vital nutrients for livestock, and the demand for both varies between different genders. The gut microbiota plays an important role in the host’s nutrient utilization and immunity. This study focuses on the effects of hindgut microbiota and metabolism on female and male Leizhou goats when consuming a 10% crude protein diet at four energy levels. It concluded that female and male goats exhibit distinct metabolic abilities when consuming a 10% crude protein diet at four different energy levels, providing a gender-based reference basis for formulating low-protein dietary strategies in goats.

## 1. Introduction

Ruminants primarily rely on a diverse and robust bacterial community for the efficient digestion of dietary fibers, which subsequently produce volatile fatty acids, microbial proteins, ammonia, and other metabolites essential for growth and reproduction [1,2]. In addition, gut bacterial communities play a vital role in the overall feed digestion and nutrient absorption in ruminants [3,4]. A number of factors influence bacterial communities’ composition. A previous study showed that the relative abundances (RAs) of Bacteroidota, Verrucomicrobiota, Cyanobacteria, *Prevotella*, and *Lachnospiraceae_NK4A136_ group* were lower, while Firmicutes and Desulfobacterota, and *Ruminococcus*, *Saccharofermentans*, *Succiniclasticum*, *Christensenellaceae_R_7_group*, and *Candidatus_Sacch-arimonas* were greater in female compared to male Qinchuan cattle [5]. Similarly, Guo et al. reported that Fibrobacteres and Spirochaetes had significantly greater RAs in female than in male Tibetan goats [6]. At the genus level, the RAs of *Fibrobacter*, *Ruminococcus_1*, and *Pyramidobacter* were significantly higher in female than in male Tibetan goats. These studies provided a gender reference for ruminants based on their bacterial communities.

Dietary composition and nutritional levels strongly influence ruminant gut microbial communities and their diversity, richness, and evenness. Wang et al. reported that rumen bacterial diversity decreased with increasing dietary energy levels in sheep, with changes in the abundance of some bacteria (e.g., *Prevotellaceae*, *Muribaculaceae*, *Saccharofermentans*, *Prevotella*, and *Succiniclasticum*) [7]. Liu et al. observed that a rumen-protected fat supplement (a high-energy additive) reduced the RAs of *Ruminococcus*, *Rikenellaceae_RC9_gut_group*, *Treponema*, *norank_f_norank_o_RF39*, *Eubacterium_siraeum_group*, and *Ruminococcus_torques_group* and increased the RAs of *Bacteroides*, *norank_f_norank_o_Clostridia_UCG-014*, *norank_f_Eubacterium_coprostanoligenes_group*, *Eubacterium_ruminantium_group*, *norank_f_Oscillospirale-UCG-010*, *Oscillospiraceae_UCG-002*, and *Family_XIII_AD3011_ group* [8]. In dairy cows, Wei et al. reported that increasing dietary crude protein (CP) increased the RA of *Paeniclostridium* and decreased the RA of *Lachnospiraceae_NK3A20_group* [9]. A previous study reported no difference in the ACE, Chao, Shannon, Simpson, and Sobs indices in goats when they consumed rumen-protected fat [8]. However, the Chao, Shannon, and Sobs indices and coverage were greater in dairy cows when they consumed a low-protein diet with rumen-protected lysine than without rumen-protected lysine [9]. Over the past few decades, scientists have mainly focused on rumen microorganisms. However, the hindgut serves as a secondary fermentation chamber in ruminants, where approximately 17% of digestible fiber is fermented, producing substantial amounts of short-chain fatty acids, including acetate, propionate, and butyrate—significant energy sources for the host. Hence, in the present study, we will focus on the fecal bacterial communities of ruminants consuming low-protein diets with varying energy levels.

Metabolomics provides a comprehensive readout of the host–microbiome metabolic interface under dietary interventions. By profiling small-molecule metabolites and pathways, metabolomics complements sequencing-based microbiome studies, revealing functional consequences of microbial shifts [10]. For instance, it has been reported that the microbiome and both its own and the host’s metabolome can regulate growth performance and production in dairy goats [11], sheep [12], crossbred cattle [13], and dairy cows [14]. Zhu et al. reported that the levels of 78 metabolites in the rumen fluid of Anhui white goats were altered (74 decreased, 4 increased) by dietary CP levels, and most of those at low-protein levels were substrates for microbial protein synthesis [15]. Ma et al. identified 14 metabolites that were consistently altered in Saanen goats when consuming a high-concentrate diet with a concentrate-to-forage ratio of 70:30 compared to the control diet with a ratio of 30:70. These spanned carbohydrate metabolism, amino acids, fatty acids, biogenic amines, and organic acids [16]. In the present study, metabolomics was also used to better reveal the effect of dietary energy levels on fecal microbiota in Leizhou goats when consuming a low-protein diet.

The Leizhou goat, also known as the Hainan black goat, is an indigenous tropical breed from southern China. It is well-adapted to hot, humid environments and produces exceptionally high-quality meat containing more than 22.6% protein, including most essential amino acids, while being low in fat (<3%) and cholesterol (<60 mg/kg) [17,18,19]. In addition, Hainan black goat meat is regarded as one of the “four famous foods” in Hainan [20]. Despite these desirable traits, the average growth performance and carcass yield of Leizhou goats remain low relative to market demand. Numerous studies have shown that increasing dietary energy levels can improve the average daily gain of Leizhou goats [8], grazing lactating yaks [21], and Hulunbuir lambs [22]. Dietary energy level and gender can affect the fecal microbiota; however, little is known about how their interaction influences the fecal microbiota in goats. The primary objective of this study was to compare the fecal microbiota and metabolomic profiles of male and female Leizhou goats fed a 10% crude protein diet with four energy levels.

## 2. Materials and Methods

### 2.1. Animal, Experimental Design, and Treatments

Eight Leizhou goats (8 months of age; four non-pregnant females and four males) were used in the experiment, with an average body weight of 10.3 ± 0.8 kg for females and 13.6 ± 1.1 kg for males (mean ± SD). Animals were assigned to two separate 4 × 4 Latin square designs based on gender, each consisting of four treatments and four 28-day periods, including 25 days of adaptation and 3 days of fecal sample collection per period. The goats were individually housed in metabolic cages equipped with feed bunkers and individual water troughs. Prior to the experimental trial, all goats underwent a 14-day adaptation period. All goats were fed a low-protein total mixed ration with four different energy levels: 7.01 (low energy, **LE**), 8.33 (middle–low energy, **MLE**), 9.66 (middle–high energy, **MHE**), and 10.98 (high energy, **HE**) MJ/kg DM. Respectively, the diets provided 0.85, 1.00, 1.15, and 1.30 times the maintenance energy requirement of goats according to the Nutrient Requirements of Meat-Type Sheep and Goats (NY/T 816-2021) [23]. The dietary ingredients and chemical composition of the experimental diets are shown in Supplementary Table S1. The four diets were weighed and provided to the goats twice daily (at 08:00 and 16:30) throughout the experimental period, alongside free access to feed and water.

### 2.2. Sample Collections

Before the morning feeding from d 26 to d 28 in each period, the fecal samples (approximately 5 g) were collected from the rectums of each goat following the method described by Wuytack et al. [24]. The fecal samples were collected into a 10 mL centrifuge tube, frozen in liquid nitrogen immediately, and stored at −80 °C until further omics analysis.

### 2.3. Fecal Bacterial Communities’ Analysis

The fecal samples underwent bacterial community analysis at Majorbio Bio-Pharm Technology Co., Ltd. (Shanghai, China). Genomic DNA was extracted from the specimens using a commercial fecal DNA extraction kit (product number: DP328, Tiangen Biotech, Beijing, China) according to the manufacturer’s protocols. The DNA quality control included concentration measurement using a Nanodrop spectrophotometer (Thermo Scientific, Wilmington, DE, USA) and integrity assessment using the FlashGel system (Lonza, Rockland, Inc., Pessac, France). Subsequent amplification targeted the V3–V4 regions of bacterial 16S rRNA using primers 338/806R under standard PCR conditions [8], with qualified PCR products sequenced on the Illumina MiSeq platform (2 × 300 paired-end sequencing runs).

For V3 and V4 amplification, the primer pair 338F (5′-ACTCCTACGGGAGGCAGCAG-3′) and 806R (5′-GGACTACHVGGGTWTCTAAT-3′) was employed. Reactions utilized TransStart FastPfu DNA polymerase (TransGen Biotech, Beijing, China) in an ABI geneAmp&reg (product number: 9700; Applied Biosystems, Foster City, CA, USA), with PCR reaction conditions as follows: 95 °C for 3 min, then 27 cycles at 95 °C for 30 s, 55 °C for 30 s, and 72 °C for 45 s, with a final extension at 72 °C for 10 min, and 10 °C until halted by the user. Triplicate 20-uL reactions contained 10 μL of 2 × Pro Taq, 0.8 μL of each primer (5 μM), 0.4 μL of FastPfu Polymerase, and 10 ng/uL of template DNA, with ddH_2_O added until 20 µL was reached. The amplified products were gel-purified (2% agarose) using the AxyPrep DNA Gel Extraction Kit (Axygen, Union City, NJ, USA) and then quantified using QuantiFluor-ST (Promega, Madison, WI, USA).

Bioinformatic processing of sequencing raw data was performed using the QIIME package (V 1.91) following established methods [25]. Raw sequences underwent adapter trimming and ambiguous read removal, followed by FLASH assembly (overlap > 10 bp; mismatch < 20%) and UCHIME-based chimera removal. The OTU clustering at 97% similarity used USEARCH, with taxonomic classification via RDP classifier (v11.5) against the Silva 138.2 database. To study the species diversity of the 32 samples, alpha diversity was used on six indices, including Sobs, Shannon, Simpson, ACE, Chao, and Good-coverage. The six indices of the 32 samples were calculated using QIIME (Quantitative Insights into Microbial Ecology) (Version 1.7.0). Linear discriminant analysis effect size (LEfSe) was determined for bacterial difference analysis in fecal bacteria between gender and among energy levels by coupling the Kruskal–Wallis Test for statistical significance with additional tests assessing biological consistency and effect relevance. Linear discriminant analysis scores were used to estimate the influence of the abundance of each species on the differential effect, using the free online Majorbio Cloud Platform (www.Majorbio.com, accessed on 18 May 2025) to generate graphical visualizations. Taxa with an LDA Score > 3 were considered as exhibiting a significant effect size.

### 2.4. Metabolites Extraction, UHPLC-MS/MS, and Metabolomic Analysis

Prior to the metabolomics profiling, the fecal samples were cryopreserved at −80 °C at Majorbio Bio-Pharm Technology Co., Ltd. (Shanghai, China). Aliquots (50.0 ± 0.1 mg) of fecal sample were homogenized in a 2-mL centrifuge tube containing a 6 mm diameter grinding bead, before adding 400 μL of ice-cold methanol:water (*v*:*v*, 4:1) extraction solvent. Mechanical disruption was performed using a high-throughput tissue grinder at a low temperature (60 Hz, −20 °C). Subsequent processing involved: (1) vortex-assisted low-temperature ultrasonication (40 kHz, 5 °C) for 30 min; (2) thermal equilibration at −20 °C for 30 min to maximize metabolite solubility; (3) centrifuged at 13,000× *g* for 15 min (4 °C); (4) the supernatant was filtered using 0.22-μm nylon membranes. Processed extracts were stored at −80 °C until LC-MS analysis.

The chromatographic separation was achieved using an ACQUITY HSS T3 column (100 mm × 2.1 mm, 1.8 μm; Waters, Milford, CT, USA) on an AB SCIEX UPLC-TripleTOF system. The mobile phases consisted of solvent A, which was mixed with 0.1% formic acid in water: acetonitrile according to a volume ratio of 95:5, and solvent B, which was mixed with 0.1% formic acid in acetonitrile: isopropanol:water following a volume ratio of 47.5:47.5. The flow rate was kept at 0.40 mL/min and the column temperature was set at 40 °C.

Mass spectrometry was conducted using a Thermo UHPLC-Q Exactive system (Thermo Fisher Scientific Inc., Beijing, China) with a dual-polarity electrospray ionization (ESI) source. The optimal conditions were set as follows: the heater temperature was 400 °C; capillary temperature was 320 °C; sheath gas flow rate, 40 arb; aux gas flow rate, 10 arb; ion-spray voltage floating (ISVF), −2800 V in negative mode and 3500 V in positive mode; and normalized collision energy, 20–40–60 V rolling for MS/MS. The total MS resolution was 70,000, and MS/MS resolution was 17,500. The data-dependent acquisition (DDA) mode was adopted for data acquisition. The detection was carried out within a mass range from 70 to 1050 *m*/*z*.

To monitor instrumental stability, pooled quality control (QC) samples were generated by combining equal-volume aliquots from all experimental samples. QC injections were interspersed at 8–10 sample intervals throughout the analytical sequence, enabling systematic evaluation of retention time shifts (<0.1 min), signal drift (RSD < 15%), and feature reproducibility. Principal component analysis (PCA) and partial least squares discriminant analysis (PLS-DA) were conducted using the multivariate statistical analysis module. Volcano plots were created using the differential analysis module, and differentially abundant metabolites were identified using *t*-tests and Variable Importance in Projection (VIP) scores (*p* < 0.05, VIP scores > 1). The differentially abundant metabolites were then subjected to Kyoto Encyclopedia of Genes and Genomes (KEGG) enrichment analysis, and the biological pathway most relevant to the experimental treatment was obtained using Fisher’s exact test. All metabolites were analyzed by Shanghai Majorbio Bio-pharm Technology Co., Ltd. (Shanghai, China) and the data were analyzed using the free online Majorbio Cloud Platform (www.Majorbio.com, accessed on 18 May 2025).

### 2.5. Statistics Analysis

The data were first organized using Excel 2023. Subsequent statistical analyses were performed using the SAS statistical package (SAS version 9.4, SAS Inst. Inc., Cary, NC, USA). The model was: Y = μ + EL + P + G + e, where Y = dependent variable; μ = treatment mean value; EL = effect of dietary energy level; P = period; G = effect of gender; and e = residual error. Polynomial contrasts were conducted to determine the effects of the increasing energy fiber level on fecal bacterial communities at genus (RA > 0.50%) and phylum (RA > 0.50%) using the SAS statistical package (SAS version 9.4, SAS Inst. Inc., Cary, NC, USA). Data were presented as mean ± standard error of the mean (SEM). Pearson’s correlation analysis was used to assess the relationship between fecal microbial taxa and metabolites. Statistical significance was set at *p* < 0.05.

## 3. Results

### 3.1. Sequencing Metrics for the Fecal Microbiota of Goats

**Figure 1:**
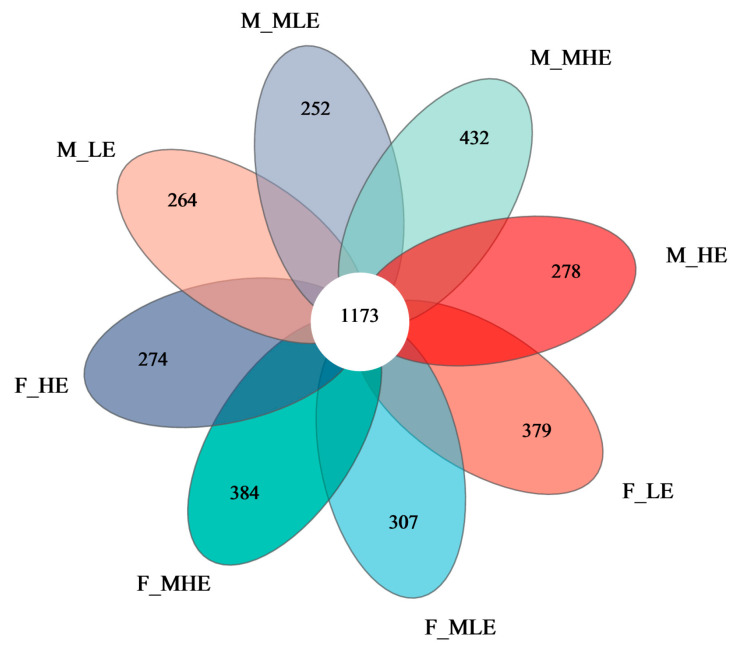
Venn diagram of OTUs of fecal microbial communities between female and male goats when consuming a 10% crude protein diet at 4 energy levels. **F-LE**, female goats fed a low energy diet (=7.01 MJ/kg DM) group (*n* = 4); **F-MLE**, female goats fed a middle–low energy diet (=8.33 MJ/kg DM) group (*n* = 4); **F-MHE**, in the female goats fed a middle–high energy diet (=9.66 MJ/kg DM) group (*n* = 4); **F-HE**, female goats fed a high energy diet (=10.98 MJ/kg DM) group (*n* = 4); **M-LE**, male goats fed a low energy diet (=7.01 MJ/kg DM) group (*n* = 4); **M-MLE**, male goats fed a middle–low energy (=8.33 MJ/kg DM) diet group (*n* = 4); **M-MHE**, male goats fed a middle–high energy diet (=9.66 MJ/kg DM) group (*n* = 4); **M-HE**, male goats fed a high energy diet (=10.98 MJ/kg DM) group(*n* = 4).

A total of 2,253,409 raw sequences were generated from 32 fecal samples, consisting of 1,163,748 sequences from female goats and 1,089,661 sequences from male goats. Based on 97% nucleotide sequence identity, 24,525 OTUs were obtained. The flower plot shows that 1173 OTUs were shared across the eight groups, accounting for 37.8%, 38.7%, 36.1%, and 42.5% of the total OTUs in the female goats and 40.2%, 38.2%, 35.1%, and 32.4% in the male goats receiving diets with 7.01, 8.33, 9.66, and 10.98 MJ/kg DM, respectively. Additionally, the numbers of specific OTUs in the LE, MLE, MHE, and HE groups were 379, 307, 384, and 274 for female goats and 264, 252, 432, and 278 for male goats, respectively (Figure 1).

**Figure 2:**
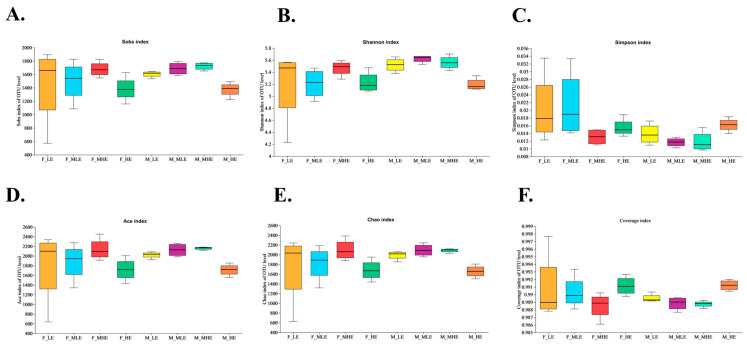
Comparison of diversity score indices of fecal bacteria in goats when consuming a 10% crude protein diet at 4 energy levels. (**A**) Sobs index in the fecal bacteria; (**B**) Shannon index in the fecal bacteria; (**C**) Simpson index in the fecal bacteria; (**D**) Ace index in the fecal bacteria; (**E**) Chao index in the fecal bacteria (**F**) Coverage index in the fecal bacteria. **F-LE**, female goats fed a low energy diet (=7.01 MJ/kg DM) group (*n* = 4); **F-MLE**, female goats fed a middle–low energy diet (=8.33 MJ/kg DM) group (*n* = 4); **F-MHE**, in the female goats fed a middle–high energy diet (=9.66 MJ/kg DM) group (*n* = 4); **F-HE**, female goats fed a high energy diet (=10.98 MJ/kg DM) group (*n* = 4); **M-LE**, male goats fed a low energy diet (=7.01 MJ/kg DM) group (*n* = 4); **M-MLE**, male goats fed a middle–low energy (=8.33 MJ/kg DM) diet group (*n* = 4); **M-MHE**, male goats fed a middle–high energy diet (=9.66 MJ/kg DM) group (*n* = 4); **M-HE**, male goats fed a high energy diet (=10.98 MJ/kg DM) group(*n* = 4).

The Shannon index was lower (*p* = 0.041) and the Simpson index was greater (*p* = 0.024) in the female goats than in the male goats. The Sobs (*p* = 0.035), ACE (*p* = 0.030), and Chao indices (*p* = 0.027) increased quadratically with rising dietary energy levels (Figure 2).

A total of 18 phyla were identified. Across all samples, Firmicutes was the most abundant phylum, with relative abundances of 71.1%, 82.7%, 78.2%, and 70.6% in the female goats, and 70.3%, 78.7%, 77.4%, and 71.5% in the male goats for diets containing 7.01, 8.33, 9.66, and 10.98 MJ/kg DM, respectively (Table 1). Bacteroidota was the second most abundant phylum with relative abundances of 21.9%, 14.7%, 18.2%, and 21.2% in the female goats and 24.8%, 17.0%, 17.7%, and 21.7% in the male goats for diets containing 7.01, 8.33, 9.66, and 10.98 MJ /kg DM, respectively. There were no differences between female and male goats in terms of the relative abundances (RAs) of Firmicutes, Bacteroidota, Spirochaetota, Verrucomicrobiota, and Patescibacteria, or their interaction with energy levels. The RA of Firmicutes increased quadratically (*p* < 0.001), and that of Spirochaetota increased linearly (*p* < 0.001), whereas the RA of Bacteroidota (*p*< 0.001) and other minor phyla (*p* < 0.001) decreased quadratically with increasing dietary energy levels.

At the genus level, a total of 316 bacterial taxa were identified. The three most dominant genera were *Christensenellaceae_R-7_group*, *Oscillospiracea_UCG-005*, and *unclassified_f_Lachnospiraceae* (Table 2). For *Christensenellaceae_R-7_group*, the RAs were 7.55%, 11.72%, 14.24%, and 10.46% in the female goats, and 10.10%, 10.42%, 9.94%, and 11.36% in the male goats for diets containing 7.01, 8.33, 9.66, and 10.98 MJ/kg DM, respectively. The RA of *Oscillospiracea_UCG-005* was 11.40%, 14.00%, 8.43%, and 7.57% in the female goats, and 13.15, 13.26, 10.74, and 10.63% in the male goats for diets containing 7.01, 8.33, 9.66, and 10.98 MJ/kg DM, respectively. The RA of *unclassified_f_Lachnospiraceae* was 9.52%, 13.93%, 12.32%, and 13.43% in the female goats, and 8.06%, 9.94%, 11.28%, and 7.42% in the male goats for diets containing 7.01, 8.33, 9.66, and 10.98 MJ/kg DM, respectively. There was a significant interaction between sex and dietary energy level on the RAs of *Christensenellaceae_R-7_group*, *Oscillospiraceae_UCG-005*, *unclassified_f_Lachnospiraceae*, *norank_f_[Eubacterium]_coprostanoligenes_group*, *Rikenellaceae_RC9_gut_group*, *norank_f_Ruminococcaceae*, *Prevotellaceae_UCG-003*. The RAs of *unclassified_f_Lachnospiraceae* (*p* < 0.001), *Bacteroides* (*p* = 0.007), *norank_f_Ruminococcaceae* (*p* = 0.024), *Mediterraneibacter* (*p* = 0.001), and *norank_f_Muribaculaceae* (*p* = 0.008) were greater, whereas the RAs of *Oscillospiraceae_UCG-005* (*p* < 0.001), *Ruminococcus* (*p* = 0.035), *Monoglobus* (*p* = 0.006), *Oscillospiraceae-NK4A214_group* (*p* = 0.008), *norank_f_F082* (*p* < 0.001), and *Prevotellaceae_UCG-003* (*p* < 0.001) were lower in the female goats than in male goats. The RAs of *Oscillospiraceae_UCG-005* (*p* < 0.001), *Monoglobus* (*p* = 0.019), *Prevotellaceae_UCG-004* (*p* = 0.002), *unclassified_c_Clostridia*(*p* = 0.001), *norank_f_Ruminococcaceae* (*p* < 0.001), and others (*p* = 0.001) decreased linearly, whereas the RAs of *norank_f_[Eubacterium]_coprostanoligenes_group* (*p* < 0.001), *Ruminococcus*(*p* < 0.001), *Treponema* (*p* < 0.001), *Oscillospiraceae-UCG-002* (*p* = 0.024), *[Eubacterium]_siraeum_group* (*p* = 0.002), *norank_f_Muribaculaceae* (*p* < 0.001), and *[Eubacterium]_ruminantium_group* (*p* = 0.002) increased linearly with increasing dietary energy levels. The RAs of *Christensenellaceae_R-7_group* (*p* = 0.047), *unclassified_f_Lachnospiraceae* (*p* = 0.013), *Rikenellaceae_RC9_gut_group* (*p* = 0.009), and *norank_o_RF39* (*p* = 0.020) increased quadratically, whereas the RA of *Mediterraneibacter* (*p* = 0.018) decreased quadratically with increasing dietary energy levels.

**Figure 3:**
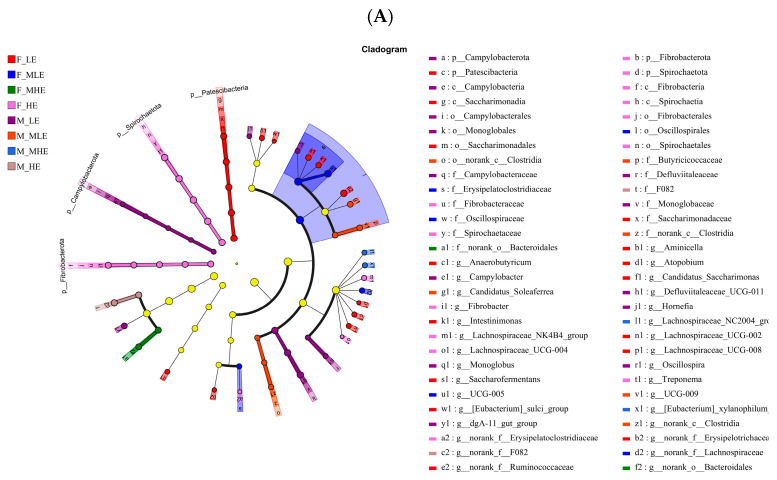

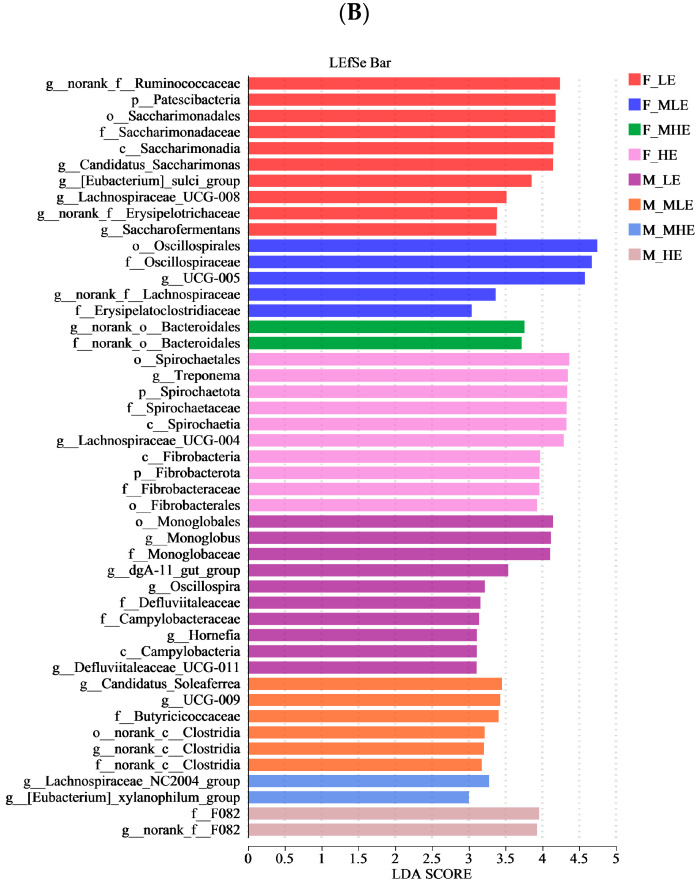
Linear discriminant analysis effect size (LEfSe) analysis. (**A**) The cladogram diagram shows the microbial species with significant differences between female and male goats when consuming a 10% crude protein diet with different energy levels. (**B**) Species with significant differences between female and male goats when consuming a 10% crude protein diet with different energy levels that have an LDA score greater than the estimated value (>2.0). Histogram bar length represents the LDA effect size; taxonomic prefixes represent classification ranks: phylum = p_, class = c_, order = o_, family = f_, and genus = g_. **F-LE**, female goats fed a low energy diet (=7.01 MJ/kg DM) group (*n* = 4); **F-MLE**, female goats fed a middle–low energy diet (=8.33 MJ/kg DM) group (*n* = 4); **F-MHE**, in the female goats fed a middle–high energy diet (=9.66 MJ/kg DM) group (*n* = 4); **F-HE**, female goats fed a high energy diet (=10.98 MJ/kg DM) group (*n* = 4); **M-LE**, male goats fed a low energy diet (=7.01 MJ/kg DM) group (*n* = 4); **M-MLE**, male goats fed a middle–low energy (=8.33 MJ/kg DM) diet group (*n* = 4); **M-MHE**, male goats fed a middle–high energy diet (=9.66 MJ/kg DM) group (*n* = 4); **M-HE**, male goats fed a high energy diet (=10.98 MJ/kg DM) group(*n* = 4).

Differential microbiota influenced by dietary energy level were further identified using linear discriminant analysis effect size (LEfSe; Figure 3A,B), with a default LDA score threshold of ±2.0. A total of 10, 5, 2, and 10 differential taxa were detected in the female goats, and 10, 6, 2, and 2 differential taxa were detected in male goats receiving diets with energy levels of 7.01, 8.33, 9.66, and 10.98 MJ/kg DM, respectively. At the genus levels, the bacteria biomarkers in the LE group were *g_norank_f_Ruminococcaceae*, *g_Candidatus_Saccharimonas*, *g_[Eubacterium]_sulci_group*, *g_Lachnospiraceae_UCG-008*, *g_norank_f_Erysipelotrichaceae*, and *g_Saccharofermentans* for female goats and *g_Monoglobus*, *g_dgA-11_gut_group*, *g_Oscillospira*, *g_Hornefia*, and *g_Defluviitaleaceae_UCG-011* for male goats. In the MLE group, the biomarkers were *g_UCG-005* and *g_norank_f_Lachnospiraceae* in the female goats, and *g_Candidatus_Soleaferra*, *g_UCG-009*, *g_norank-c_Clostridia*, and *g_norank-c_Clostridia* in male goats. In the MHE group, the biomarkers included *g_norank_o_Bacteroidales* in the female goats, and *g_Lachnospiraceae_NC2004_group* and *g_[Eubacterium]_xylanophilum_group* in male goats. In the HE group, the biomarkers were *g_Treponema* and *g_Lachnospiaceae_UCG-004* in the female goats, and *F-082* and *g_norank_f_F082* in the male goats.

### 3.2. Fecal Metabolomics Profiling Between Female and Male Goats When Consuming a Low-Protein Diet with Different Energy Levels

**Figure 4:**
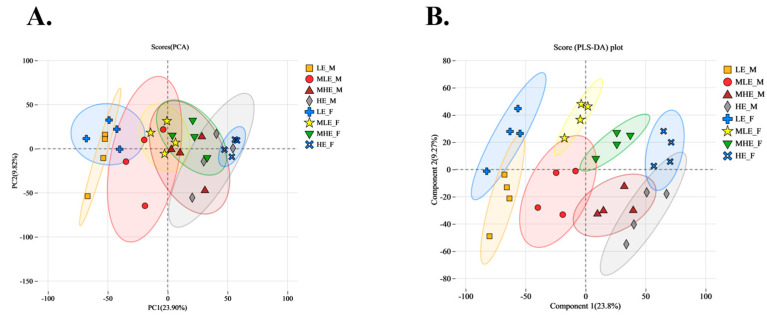
PCA (**A**) and PLA-DA (**B**) of fecal microbial metabolites in female and male goats when consuming a 10% crude protein diet with different energy levels. **LE-F**, female goats fed a low energy diet (=7.01 MJ/kg DM) group (*n* = 4); **MLE-F**, female goats fed a middle–low energy diet (=8.33 MJ/kg DM) group (*n* = 4); **MHE-F**, in the female goats fed a middle–high energy diet (=9.66 MJ/kg DM) group (*n* = 4); **HE-F**, female goats fed a high energy diet (=10.98 MJ/kg DM) group (*n* = 4); **LE-M**, male goats fed a low energy diet (=7.01 MJ/kg DM) group (*n* = 4); **MLE-M**, male goats fed a middle–low energy (=8.33 MJ/kg DM) diet group (*n* = 4); **MHE-M**, male goats fed a middle–high energy diet (=9.66 MJ/kg DM) group (*n* = 4); **HE-M**, male goats fed a high energy diet (=10.98 MJ/kg DM) group (*n* = 4).

To provide a comprehensive overview of the differences in fecal metabolite profiles between female and male goats consuming a low-protein diet with varying energy levels, principal component analysis (PCA) and partial least squares discriminant analysis (PLS-DA) were conducted. The scatterplots of both PCA and PLS-DA suggested clear separations based on gender and dietary energy levels, particularly along principal components 1 and 2, indicating substantial differences in fecal metabolomic profiles (Figure 4).

**Figure 5:**
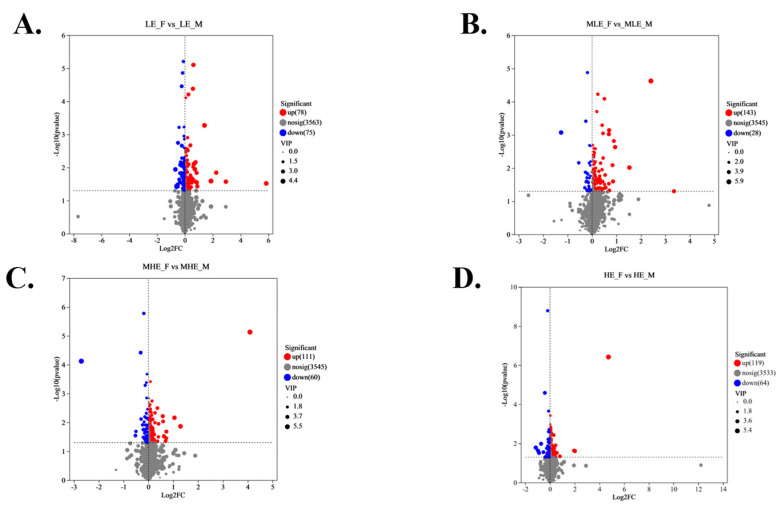
Volcano map of fecal metabolites of female and male goats when consuming a 10% crude protein diet with different energy levels. (**A**) Volcano map of fecal metabolites of female and male goats when consuming a 10% crude protein diet in the 7.01 MJ/kg group; (**B**) volcano map of fecal metabolites of female and male goats when consuming a 10% crude protein diet in the 8.33 MJ/kg group; (**C**) volcano map of fecal metabolites of female and male goats when consuming a 10% crude protein diet in the 9.66 MJ/kg group; (**D**) volcano map of fecal metabolites of female and male goats when consuming a 10% crude protein diet in the 10.98 MJ/kg group; **LE-F**, female goats fed a low energy diet (=7.01 MJ/kg DM) group (*n* = 4); **MLE-F**, female goats fed a middle–low energy diet (=8.33 MJ/kg DM) group (*n* = 4); **MHE**,-F in the female goats fed a middle–high energy diet (=9.66 MJ/kg DM) group (*n* = 4); **HE-F**, female goats fed a high energy diet (=10.98 MJ/kg DM) group (*n* = 4); **LE-M**, male goats fed a low energy diet (=7.01 MJ/kg DM) group (*n* = 4); **MLE-M**, male goats fed a middle–low energy (=8.33 MJ/kg DM) diet group (*n* = 4); **MHE-M**, male goats fed a middle–high energy diet (=9.66 MJ/kg DM) group (*n* = 4); **HE-M**, male goats fed a high energy diet (=10.98 MJ/kg DM) group (*n* = 4).

**Figure 6:**
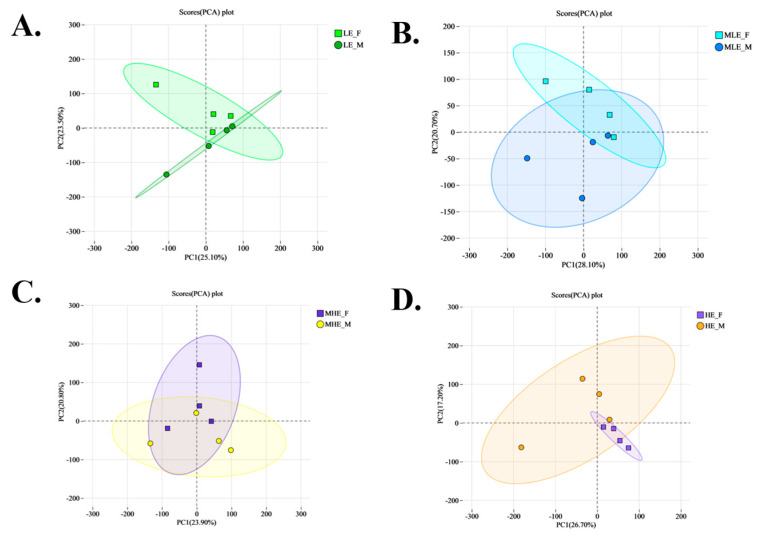
Principal component analysis (PCA) of fecal metabolites of female and male goats when consuming a 10% crude protein diet at four energy levels. (**A**) Results of PCA of fecal metabolites of female and male goats when consuming a 10% crude protein diet in the 7.01 MJ/kg group; (**B**) results of PCA of fecal metabolites of female and male goats when consuming a 10% crude protein diet in the 8.33 MJ/kg group; (**C**) results of PCA of fecal metabolites of female and male goats when consuming a 10% crude protein diet in the 9.66 MJ/kg group; (**D**) results of PCA of fecal metabolites of female and male goats when consuming a 10% crude protein diet in the 10.98 MJ/kg group; **LE-F**, female goats fed a low energy diet (=7.01 MJ/kg DM) group (*n* = 4); **MLE-F**, female goats fed a middle–low energy diet (=8.33 MJ/kg DM) group (*n* = 4); **MHE-F**, in the female goats fed a middle–high energy diet (=9.66 MJ/kg DM) group (*n* = 4); **HE-F**, female goats fed a high energy diet (=10.98 MJ/kg DM) group (*n* = 4); **LE-M**, male goats fed a low energy diet (=7.01 MJ/kg DM) group (*n* = 4); **MLE-M**, male goats fed a middle–low energy (=8.33 MJ/kg DM) diet group (*n* = 4); **MHE-M**, male goats fed a middle–high energy diet (=9.66 MJ/kg DM) group (*n* = 4); **HE-M**, male goats fed a high energy diet (=10.98 MJ/kg DM) group(*n* = 4).

**Figure 7:**
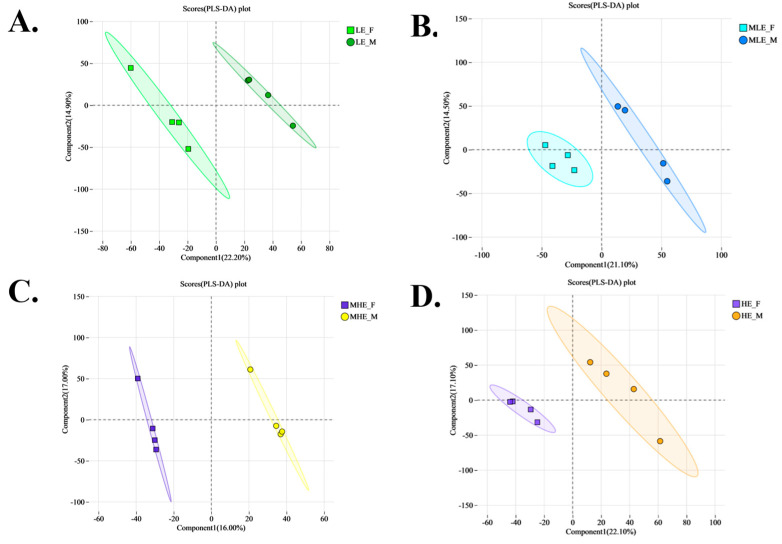
PLS-DA of fecal microbial metabolites of female and male goats when consuming a 10% crude protein diet with different energy levels. (**A**) Results of PLS-DA of fecal metabolites of female and male goats when consuming a 10% crude protein diet in the 7.01 MJ/kg group; (**B**) results of PLS-DA of fecal metabolites of female and male goats when consuming a 10% crude protein diet in the 8.33 MJ/kg group; (**C**) results of PLS-DA of fecal metabolites of female and male goats when consuming a 10% crude protein diet in the 9.66 MJ/kg group; (**D**) results of PLS-DA of fecal metabolites of female and male goats when consuming a 10% crude protein diet in the 10.98 MJ/kg group. **LE-F**, female goats fed a low energy diet (=7.01 MJ/kg DM) group (*n* = 4); **MLE-F**, female goats fed a middle–low energy diet (=8.33 MJ/kg DM) group (*n* = 4); **MHE-F**, in the female goats fed a middle–high energy diet (=9.66 MJ/kg DM) group (*n* = 4); **HE-F**, female goats fed a high energy diet (=10.98 MJ/kg DM) group (*n* = 4); **LE-M**, male goats fed a low energy diet (=7.01 MJ/kg DM) group (*n* = 4); **MLE-M**, male goats fed a middle–low energy (=8.33 MJ/kg DM) diet group (*n* = 4); **MHE-M**, male goats fed a middle–high energy diet (=9.66 MJ/kg DM) group (*n* = 4); **HE-M**, male goats fed a high energy diet (=10.98 MJ/kg DM) group(*n* = 4).

The volcano plots show that a total of 153, 171, 171, and 183 differential metabolites were identified between female and male goats fed diets containing 7.01, 8.33, 9.66, and 10.98 MJ/kg DM, respectively (Figure 5). Among these, 78, 143, 111, and 119 metabolites were upregulated, while 75, 28, 60, and 64 were downregulated at the respective energy levels. PLS-DA analysis further confirmed the distinct metabolic profiles between genders, with corresponding model performance parameters as follows: R^2^X = 0.222, R^2^Y = 0.852, and Q^2^ = 0.277, R^2^X = 0.211, R^2^Y = 0.848, and Q^2^ = 0.223, R^2^X = 0.160, R^2^Y = 0.971, and Q^2^ = 0.219, R^2^X = 0.221, R^2^Y = 0.855, and Q^2^ = 0.300 for the 7.01, 8.33, 9.66, and 10.98 MJ/kg DM, respectively (Figure 6 and Figure 7).

**Figure 8:**
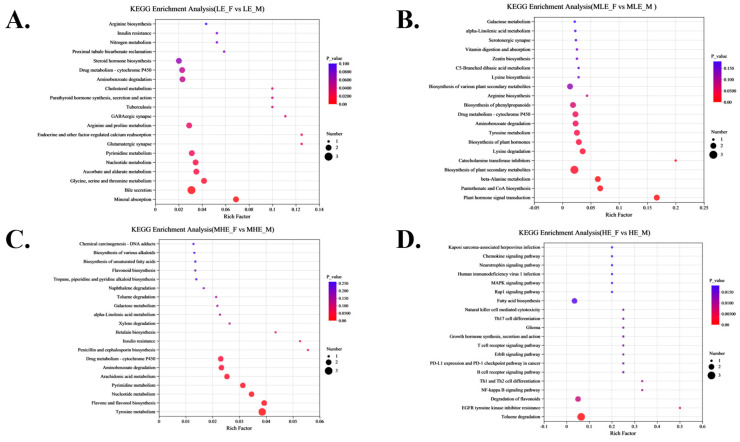
Effect of KEGG enrichment analysis on female and male goats when consuming a 10% crude protein diet with different energy levels. (**A**) Results of KEGG enrichment analysis of fecal metabolites of female and male goats when consuming a 10% crude protein diet in the 7.01 MJ/kg group; (**B**) results of KEGG enrichment analysis of fecal metabolites of female and male goats when consuming a 10% crude protein diet in the 8.33 MJ/kg group; (**C**) results of KEGG enrichment analysis of fecal metabolites of female and male goats when consuming a 10% crude protein diet in the 9.66 MJ/kg group; (**D**) results of KEGG enrichment analysis of fecal metabolites of female and male goats when consuming a 10% crude protein diet in the 10.98 MJ/kg group; **LE-F**, female goats fed a low energy diet (=7.01 MJ/kg DM) group (*n* = 4); **MLE-F**, female goats fed a middle–low energy diet (=8.33 MJ/kg DM) group (*n* = 4); **MHE-F**, in female goats fed a middle–high energy diet (=9.66 MJ/kg DM) group (*n* = 4); **HE-F**, female goats fed a high energy diet (=10.98 MJ/kg DM) group (*n* = 4); **LE-M**, male goats fed a low energy diet (=7.01 MJ/kg DM) group (*n* = 4); **MLE-M**, male goats fed a middle–low energy (=8.33 MJ/kg DM) diet group (*n* = 4); **MHE-M**, male goats fed a middle–high energy diet (=9.66 MJ/kg DM) group (*n* = 4); **HE-M**, male goats fed a high energy diet (=10.98 MJ/kg DM) group(*n* = 4).

**Figure 9:**
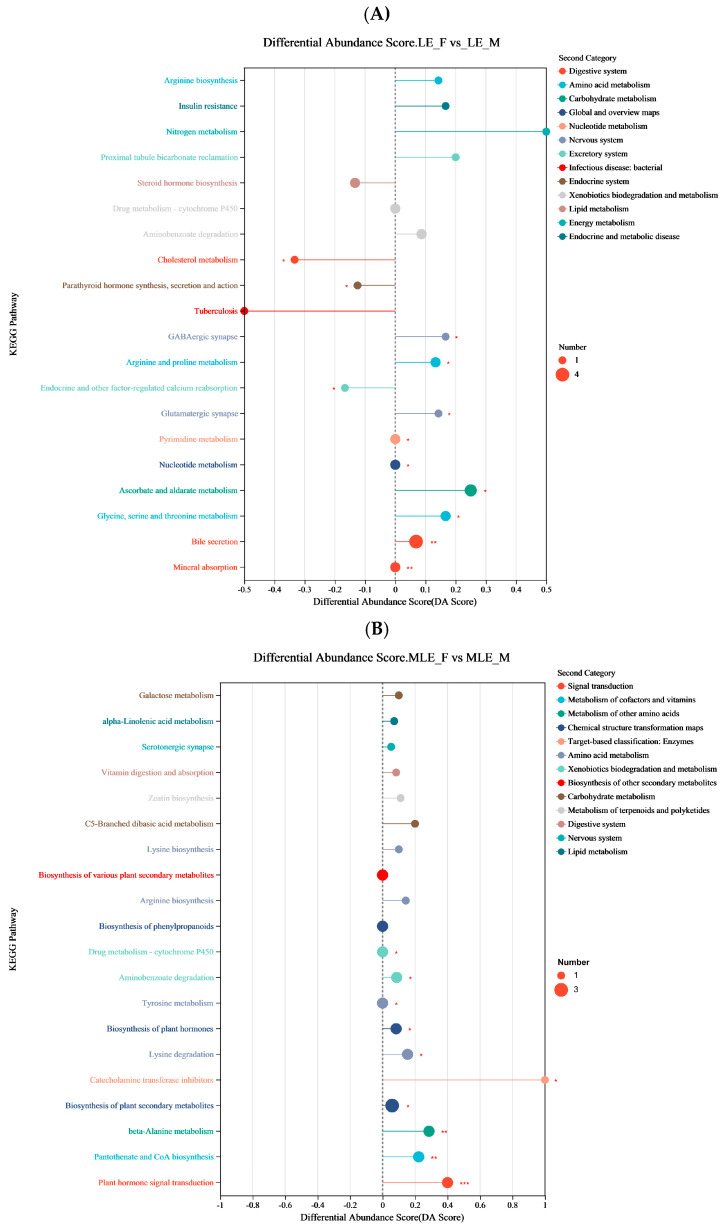

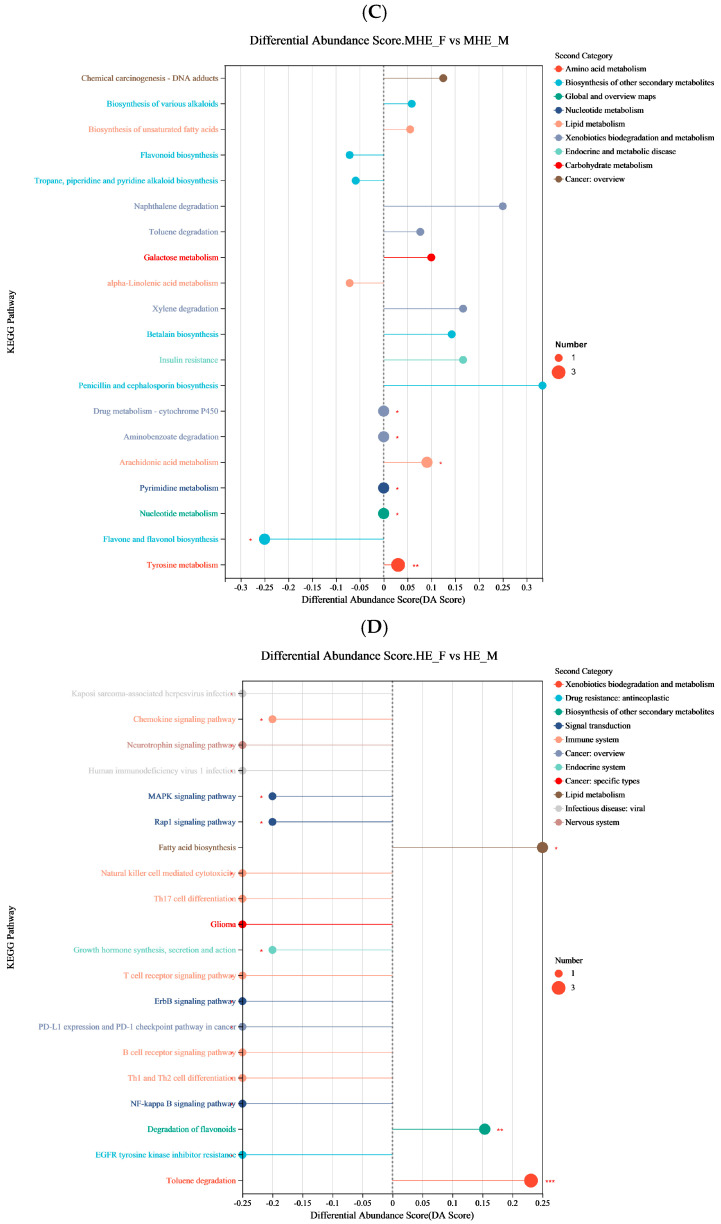
Effect of differential abundance score of KEGG of female and male goats when consuming a 10% crude protein diet with different energy levels. (**A**) Results of differential abundance score of KEGG of fecal metabolites of female and male goats when consuming a 10% crude protein diet in the 7.01 MJ/kg group; (**B**) results of differential abundance score of KEGG of fecal metabolites of female and male goats when consuming a 10% crude protein diet in the 8.33 MJ/kg group; (**C**) results of differential abundance score of KEGG of fecal metabolites of female and male goats when consuming a 10% crude protein diet in the 9.66 MJ/kg group; (**D**) results of differential abundance score of KEGG of fecal metabolites of female and male goats when consuming a 10% crude protein diet in the 10.98 MJ/kg group; **LE-F**, female goats fed a low energy diet (=7.01 MJ/kg DM) group (*n* = 4); **MLE-F**, female goats fed a middle–low energy diet (=8.33 MJ/kg DM) group (*n* = 4); **MHE-F**, in female goats fed a middle–high energy diet (=9.66 MJ/kg DM) group (*n* = 4); **HE-F**, female goats fed a high energy diet (=10.98 MJ/kg DM) group (*n* = 4); **LE-M**, male goats fed a low energy diet (=7.01 MJ/kg DM) group (*n* = 4); **MLE-M**, male goats fed a middle–low energy (=8.33 MJ/kg DM) diet group (*n* = 4); **MHE-M**, male goats fed a middle–high energy diet (=9.66 MJ/kg DM) group (*n* = 4); **HE-M**, male goats fed a high energy diet (=10.98 MJ/kg DM) group (*n* = 4). * *p* < 0.05, ** *p* < 0.01, and *** *p* < 0.001.

To further elucidate the metabolic pathways associated with sex-related differences, KEGG pathway enrichment analysis was performed on the differential metabolites between female and male goats at each energy level. The results showed that in the LE group, the differential metabolites were significantly enriched in pathways such as bile secretion, mineral absorption, glycine, serine, and threonine and nitrogen metabolism. In the MLE group, the enriched pathways included biosynthesis of plant secondary metabolites, vitamin digestion and absorption, and alpha-Linolenic acid metabolism. In the MHE group, significant enrichment was observed in pathways such as tyrosine metabolism, flavone and flavonol biosynthesis, and alpha-Linolenic acid metabolism. In the HE group, the differential metabolites were significantly enriched in pathways including toluene degradation, degradation of flavonoids, and fatty acid biosynthesis (Figure 8 and Figure 9).

### 3.3. Correlation Analysis Between Fecal Metabolites and Bacteria

**Figure 10:**
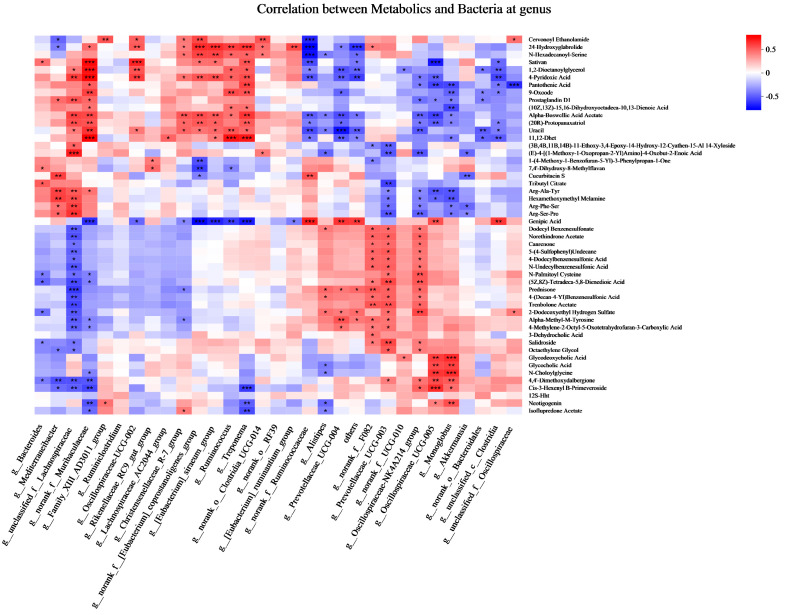
Pearson’s rank correlation analysis of the fecal bacteria (at genus, RA > 0.50%) and fecal metabolites (Top 50). * *p* < 0.05, ** *p* < 0.01, and *** *p* < 0.001 according to Pearson’s rank correlation coefficient. The red and blue colors indicate positive and negative correlations, respectively.

Correlation analyses showed numerous significant associations between differential metabolites and microbial taxa (Figure 10). Pantothenic acid was positively correlated with *norank_f_Muribaculaceae* and *Treponema*, while negatively correlated with *Oscillospiraceae-NK4A214_group*, *Oscillospiraceae_UCG-005*, *Monoglobus*, *unclassified_c_Clostridia*, and *unclassified_f_Oscillospiraceae.* Glycodeoxycholic acid showed positive correlations with *norank_f_UCG-010*, *Oscillospiraceae_UCG-005*, and *Monoglobus*. Similarly, glycocholic acid was positively correlated with *Oscillospiraceae_UCG-005* and *Monoglobus*, but negatively correlated with *Alistipes*. Uracil was positively correlated with *unclassified_f_Lachnospiraceae*, *norank_f_Muribaculaceae*, *Oscillospiraceae-UCG-002*, *Christensenellaceae_R-7_group*, *norank_f_[Eubacterium]_coprostanoligenes_group*, *[Eubacterium]_siraeum_group*, *Ruminococcus*, *Treponema*. In contrast, uracil was negatively correlated with *norank_f_Ruminococcaceae*, *Alistipes*, *Prevotellaceae_UCG-004*, *others*, *norank_f_F082*, *Prevotellaceae_UCG-003*, *norank_f_UCG-010*, *Oscillospiraceae-NK4A214_group*, *Oscillospiraceae_UCG-005*, *Monoglobus*, *Akkermansia*, *norank_o_ Bacteroidales*, and *unclassified_c_Clostridia*.

## 4. Discussion

In general, greater microbial diversity and richness are considered beneficial to host health and contribute to microbiota stability [26,27]. The richness and diversity of bacterial communities in the gastrointestinal tract can be influenced by several factors, including dietary composition and nutrient levels [28,29], as well as the age and species of animals [27,30,31]. In this study, dietary energy level and gender did not affect the Sobs, Ace, Chao, or Coverage indices of the fecal bacterial community in goats. However, the Shannon index was lower, and the Simpson index was greater in the female goats than in males. A previous study in Tibetan goats reported numerically greater Shannon and Simpson indices in females than in males [6].

At the phylum level, Firmicutes and Bacteroidota were the dominant fecal bacteria across all dietary energy levels and in both male and female goats. These results are in agreement with previous studies in Boer goats [30], Blond Adamellan goats [32], and dairy goats [33]. In the present study, there were no significant differences between female and male goats in the RAs of Firmicutes, Bacteroidota, Spirochaetota, Verrucomicrobiota, Patescibacteria, and others. In contrast, a previous study reported higher RAs of Fibrobacteres and Spirochaetes in female compared to male Tibetan goats [6]. Similarly, Pan et al. reported that the RAs of Bacteroidota, Verrucomicrobiota, and Cyanobacteria were higher, while those of Firmicutes and Desulfobacterota were lower in the rumen fluid samples of male compared to female Qinchuan cattle [5]. Such discrepancies across studies may contribute to differences in dietary composition and among animal species. In the present study, the highest RA of Firmicutes and the lowest RA of Bacteroidota were observed in the 8.33 MJ/kg DM group. This aligns with previous studies showing that bacterial communities could change in Shaanxi white cashmere goats [34], Yunshang black goat [35], and fattening male Hu lambs [36] when consuming diets with different energy levels. In the rumen ecosystem, Spirochaetes are known to degrade cellulose, pectin, and phytic acid, contributing to the utilization of fermentable carbohydrates and the production of volatile fatty acids [37]. In this study, the RA of Spirochaetota increased linearly with increasing dietary energy levels. Previous studies reported that the digestibility of neutral detergent fiber and acid detergent fiber decreased with increasing dietary energy levels in yaks [38], Tibetan sheep [39], and Small-tailed Han sheep [39]. We infer that increased fecal fiber content at higher energy levels may support the proliferation of *Spirochaetota*, explaining the observed linear increase in their abundance.

A previous study showed that *Prevotella_1* was the dominant genus in the rumen and did not differ between male and female Tibetan goats [22]. Interestingly, in the current study, *unclassified_f__Lachnospiraceae* was the dominant genus in the female goats, while *Oscillospiraceae_UCG-005* dominated in male goats. Additionally, compared with male goats, in female goats, the RA of *Oscillospiraceae_UCG-005* was lower, and that of *unclassified_f__Lachnospiraceae* was significantly greater. These differences in dominant fecal genera may be associated with dietary composition. One study reported that the RAs of *Fibrobacter*, *Ruminococcus_1*, *Erysipelotrichaceae_UCG-004, Oscillospira*, and *Pyramidobacter* were significantly higher, whereas those of *Lachnospira* and *Ruminococcaceae_NK4A214_group* were lower in female than in male Tibetan goats [22]. In the current study, the RAs of several bacterial genera also increased or decreased in a gender-dependent manner, which suggests that fecal bacterial communities respond differently between sexes even when consuming the same diet. One study reported that an increased RA of *Christensenellaceae_R-7_group* was associated with improved growth performance in goats [40]. In addition, numerous studies have shown that higher dietary energy levels enhance average daily gain in yaks [38] and Small-tailed Han sheep [39], which may partly explain the increased proportion of *Christensenellaceae_R-7_group* observed with rising dietary energy levels in the current study. The genus *Muribaculaceae_unclassified* remains relatively understudied. In the present study, the RA of *norank_f__Muribaculaceae* increased with increasing dietary energy levels. This finding is consistent with a study of Saanen dairy goats, where supplementation with 90 g of conjugated linoleic acid effectively increased dietary energy and also led to an increase in the abundance of *Muribaculaceae* [41].

Fecal metabolomics complements microbial functional genomic interpretation by reflecting the metabolic outputs of gut microbiota. The results of the present study showed that fecal metabolite profiles differed between female and male goats when fed a low-protein diet with varying energy levels. In addition, even when consuming the same diet, distinct metabolomic differences were observed between the male and female goats. The differential metabolites were primarily classified as organic acids, lipids, and amino acids. Pan et al. reported that pathways related to amino acid and lipid metabolism were mainly enriched in female Qinchuan cattle, whereas carbohydrate metabolism and glycan biosynthesis and metabolism pathways were more enriched in males [5]. Consistently, the KEGG pathway analyses performed in this study revealed gender-specific enrichment patterns under the same dietary energy levels. Specifically, the differentially enriched pathways included bile secretion, mineral absorption, glycine, serine, threonine metabolism, and nitrogen metabolism at 7.01 MJ/kg DM; biosynthesis of plant secondary metabolites, vitamin digestion and absorption, and alpha-linolenic acid metabolism at 8.33 MJ/kg DM; tryosine metabolism, flavone and flavonol biosynthesis and alpha-Linolenic acid metabolism at 9.66 MJ/kg DM; and toluene degradation, degradation of flavonoids, and fatty acid biosynthesis at 10.98 MJ/kg DM. These findings suggest that gender influences fecal metabolite profiles by modulating metabolism pathways related to amino acid metabolism, as well as cofactors and vitamin metabolism.

Pantothenic acid, an essential nutrient, plays a vital role in metabolism as a component of coenzyme A and acyl-carrier-proteins [42,43]. Yi et al. reported that pantothenic acid concentrations were downregulated in animals fed a concentrate-to-forage ratio of 65:35 compared to those fed a 50:50 ratio, and found no correlation between rumen bacterial genera and pantothenic acid concentrations [44]. However, Yoshii et al. [43] reported that several intestinal commensal bacteria possess pantothenic acid biosynthesis pathways, including Bacteroides *fragilis* and *Prevotella copri* (Bacteroidetes), some *Ruminococcus spp.* (*R. lactaris* and *R. torques*) (*Firmicutes*), and *Salmonella enterica* and *Helicobacter pylori* (*Proteobacteria*), indicating their capacity to produce pantothenic acid endogenously. Our results showed that pantothenic acid was positively correlated with *norank_f_Muribaculaceae* and *Treponema* and negatively correlated with *Oscillospiraceae-NK4A214_group*, *Oscillospiraceae_UCG-005*, *Monoglobus*, *unclassified_c_Clostridia*, and *unclassified_f_Oscillospiraceae.* These findings suggest a selective association between pantothenic acid levels and specific microbial taxa in the goat hindgut. Previous studies have shown that glycodeoxycholic acid and glycocholic acid can alter bile acid metabolism and reshape intestinal microbiota [45]. We consistently found that glycodeoxycholic acid was positively associated with *norank_f_UCG-010*, *Oscillospiraceae_UCG-005*, and *Monoglobus*. Glycocholic acid was positively correlated with *Oscillospiraceae_UCG-005* and *Monoglobus*, but negatively correlated with *Alistipes*. Uracil, which may serve as a microbial genetic marker in ruminants, is also implicated in enhancing microbial protein synthesis. Zhang et al. demonstrated that increased synchronization energy and nitrogen release in the rumen enhanced microbial protein synthesis [46], accompanied by an increased abundance of *Fibrobacter* and *Ruminobacter* and decreased levels of *Klebsiella* and *Succinivibrio*. In the present study, uracil was positively correlated with *Oscillospiraceae-UCG-002*, *Christensenellaceae_R-7_group*, *norank_f_[Eubacterium]_coprostanoligenes_group*, *[Eubacterium]_siraeum_group*, *Ruminococcus*, and *Treponema*. In contrast, negative correlations were observed with *norank_f_Ruminococcaceae*, *Alistipes*, *Prevotellaceae_UCG-004*, *norank_f_F082*, *Prevotellaceae_UCG-003*, *norank_f_UCG-010*, *Oscillospiraceae-NK4A214_group*, *Oscillospiraceae_UCG-005*, *Monoglobus*, and *Akkermansia*. Taken together, these findings suggest that uracil has a substantial impact on the gut microbial ecosystem and serves as a key indicator of fecal metabolic profiles in response to dietary and host-related factors.

## 5. Conclusions

This study investigated the effects of gender and dietary energy level on fecal metabolite profiles and microbiota composition in goats fed a low-protein diet. The results showed that *unclassified_f_Lachnospiraceae* was the dominant genus in female goats, while *Oscillospiraceae_UCG-005* was the dominant genus in male goats. Additionally, distinct differences in fecal metabolite profiles were observed between female and male goats under varying dietary energy levels. It is concluded that the female and male goats exhibit different metabolic responses to diets containing 10% crude protein with varying energy levels. These findings provide a basis for developing gender-specific dietary guidelines for 8-month-old Leizhou goats under low-protein feeding conditions.

## Figures and Tables

**Table 1:** Relative abundance of fecal bacterial community at phylum level (0.5% < RA) in goats when consuming a 10% crude protein diet at 4 energy levels.

Items	Gender	Dietary Energy Levels				SEM	*p*-Values				
		LE	MLE	MHE	HE		G	E	G × E	E-L	E-Q
Firmicutes	Female	71.1	82.7	78.2	70.6	0.93	0.285	<0.001	0.427	0.691	<0.001
	Male	70.3	78.7	77.4	71.5						
Bacteroidota	Female	21.9	14.7	18.2	21.2	0.71	0.223	<0.001	0.621	0.439	<0.001
	Male	24.8	17.0	17.7	21.7						
Spirochaetota	Female	0.46	0.92	1.79	3.94	0.234	0.126	<0.001	0.059	<0.001	0.316
	Male	0.69	2.12	2.49	3.30						
Verrucomicrobiota	Female	0.57	0.31	0.52	1.44	0.202	0.285	0.418	0.211	0.649	0.146
	Male	2.23	0.72	0.94	0.67						
Patescibacteria	Female	2.55	0.42	0.09	0.08	0.274	0.365	0.130	0.603	0.038	0.213
	Male	0.80	0.24	0.09	0.05						
Others	Female	3.34	0.90	1.17	2.82	0.195	0.128	<0.001	0.008	0.245	<0.001
	Male	1.21	1.27	1.41	2.71						

**LE** = low energy group, 7.01 MJ/kg DM; **MLE** = middle–low energy group, 8.33 MJ/kg DM; **MHE** = middle–high energy group, 9.66 MJ/kg DM; **HE** = high energy group, 10.98 MJ/kg DM; **SEM** = standard error of the means. G = effect of female and male; E = effect of different energy levels; G × E= effect of interaction between G and E; E-L = Linear effect of dietary energy levels; E-Q = Quadratic effect of dietary energy levels.

**Table 2:** Relative abundance of fecal bacterial community at genus level (0.5% < RA) in goats when consuming a 10% crude protein diet at 4 energy levels.

Items	Gender	Dietary Energy Levels				SEM	*p*-Values				
		LE	MLE	MHE	HE		G	E	G × E	E-L	E-Q
*unclassified_f__* *Lachnospiraceae*	Female	9.52	13.93	12.32	13.42	0.46	<0.001	0.001	0.008	0.223	0.013
	Male	8.06	9.94	11.28	7.41						
*Oscillospiraceae* *_UCG-005*	Female	11.40	14.00	8.43	7.57	0.42	<0.001	<0.001	0.005	<0.001	0.086
	Male	13.15	13.26	10.74	10.63						
*Christensenellacea* *e_R-7_group*	Female	7.55	11.72	14.24	10.46	0.45	0.459	0.026	0.015	0.059	0.047
	Male	10.10	10.42	9.94	11.36						
*norank_f__[Eubacterium]_* *coprostanoligenes_group*	Female	3.11	5.70	10.56	6.28	0.48	0.086	<0.001	0.007	<0.001	0.002
	Male	4.51	6.30	8.67	9.53						
*Rikenellaceae_RC9* *_gut_group*	Female	6.53	7.11	5.37	7.50	0.25	0.913	0.014	0.002	0.515	0.009
	Male	8.45	4.83	6.49	6.58						
*Ruminococcus*	Female	1.86	4.64	3.79	4.97	0.29	0.035	<0.001	0.446	<0.001	0.232
	Male	2.49	4.64	4.64	6.58						
*norank_o__Clostridia_* *UCG-014*	Female	2.28	3.28	3.93	3.96	0.30	0.379	0.265	0.457	0.327	0.101
	Male	3.03	5.21	4.06	3.25						
*Bacteroides*	Female	4.80	3.05	6.07	5.91	0.31	0.007	0.045	0.059	0.856	0.278
	Male	4.79	3.00	3.59	2.79						
*Monoglobus*	Female	2.44	2.15	2.99	1.94	0.22	0.006	0.003	0.073	0.019	0.060
	Male	4.08	3.87	3.90	1.46						
*Prevotellaceae_UCG* *-004*	Female	4.56	1.35	2.27	1.67	0.29	0.726	0.008	0.325	0.002	0.235
	Male	3.74	3.10	2.27	1.45						
*Treponema*	Female	0.46	0.91	1.77	3.90	0.24	0.178	<0.001	0.103	<0.001	0.385
	Male	0.68	2.12	2.46	3.23						
*norank_f__UCG-010*	Female	1.71	1.91	2.46	1.46	0.12	0.395	0.326	0.554	0.455	0.125
	Male	2.22	2.24	2.07	1.82						
*Akkermansia*	Female	0.25	0.26	0.41	1.39	0.20	0.352	0.503	0.123	0.745	0.168
	Male	2.06	0.62	0.60	0.48						
*unclassified_c__* *Clostridia*	Female	2.62	2.09	1.24	0.73	0.22	0.950	0.013	0.769	0.001	0.419
	Male	2.03	2.70	1.22	0.62						
*Alistipes*	Female	2.12	0.98	0.77	2.08	0.14	0.665	0.068	0.173	0.272	0.017
	Male	2.02	1.63	1.45	1.30						
*norank_o__RF39*	Female	0.90	1.43	1.37	1.65	0.10	0.944	0.039	0.064	0.160	0.020
	Male	0.96	1.44	2.04	0.86						
*Lachnospiraceae_* *AC2044_group*	Female	0.85	1.86	1.44	1.89	0.13	0.372	0.764	0.196	0.420	0.679
	Male	1.48	1.12	1.38	1.14						
*norank_f__Rumin-* *ococcaceae*	Female	3.75	1.34	0.69	0.86	0.19	0.024	<0.001	0.002	<0.001	0.003
	Male	1.67	1.15	0.66	1.18						
*Oscillospiraceae-NK4A214_group*	Female	1.35	0.89	1.00	0.99	0.06	0.008	0.143	0.524	0.231	0.084
	Male	1.43	1.32	1.18	1.40						
*Oscillospiraceae-* *UCG-002*	Female	0.68	0.99	1.11	1.90	0.12	0.999	0.170	0.473	0.024	0.796
	Male	0.97	1.00	1.45	1.25						
*norank_f__F082*	Female	0.85	0.25	0.31	0.53	0.14	0.001	0.098	0.134	0.219	0.111
	Male	0.96	0.71	1.35	2.06						
*Mediterraneibacter*	Female	1.52	0.99	0.59	1.24	0.08	0.001	0.018	0.154	0.767	0.018
	Male	0.55	0.55	0.44	0.85						
*Prevotellaceae_UCG-003*	Female	0.06	0.02	0.02	0.07	0.10	<0.001	<0.001	<0.001	0.145	0.050
	Male	0.64	0.64	0.19	1.59						
*[Eubacterium]_siraeum* *_group*	Female	0.32	0.72	0.85	0.89	0.05	0.790	0.004	0.360	0.002	0.017
	Male	0.52	0.81	0.85	0.69						
*unclassified_f__Oscill-ospiraceae*	Female	0.81	0.66	0.61	0.55	0.06	0.551	0.521	0.983	0.153	0.729
	Male	0.87	0.67	0.75	0.62						
*g__Family_XIII_* *AD3011_group*	Female	0.58	0.65	0.87	0.53	0.04	0.810	0.300	0.718	0.812	0.152
	Male	0.67	0.62	0.69	0.56						
*norank_o__Bacteroidales*	Female	1.01	0.15	1.37	0.51	0.11	0.263	0.077	0.108	0.261	0.468
	Male	0.97	0.46	0.29	0.40						
*Ruminiclostridium*	Female	0.72	2.56	0.32	0.11	0.21	0.202	0.080	0.102	0.348	0.063
	Male	0.12	0.51	0.91	0.19						
*norank_f__Muriba-* *culaceae*	Female	0.25	0.80	0.67	1.62	0.09	0.008	<0.001	0.133	<0.001	0.718
	Male	0.10	0.64	0.52	0.85						
*[Eubacterium]_ruminan* *tium_group*	Female	0.17	0.30	0.72	0.72	0.07	0.213	0.004	0.696	0.002	0.296
	Male	0.41	0.40	1.04	0.67						
*others*	Female	24.96	13.31	11.44	12.71	0.80	0.487	<0.001	<0.001	0.001	<0.001
	Male	16.27	14.06	12.86	17.15						

**LE** = low energy group, 7.01 MJ/kg DM; **MLE** = middle–low energy group, 8.33 MJ/kg DM; **MHE** = middle–high energy group, 9.66 MJ/kg DM; **HE** = high energy group, 10.98 MJ/kg DM.; **SEM** = standard error of the means. G = effect of female and male; E = effect of different energy levels; G × E = effect of interaction between G and E; E-L = linear effect of dietary energy levels; E-Q= quadratic effect of dietary energy levels.

## Data Availability

Data may be provided upon request to the corresponding author (liuh2018@lzu.edu.cn).

## Supplementary Materials

The following supporting information can be downloaded at: https://www.mdpi.com/article/10.3390/ani15152174/s1, Table S1: Composition of dietary ingredients and nutritional levels of the 4 treatments.
