# Supplementary material for: Comparative Analysis of Fecal Microbiota and Metabolomic Profiles in Male and Female Leizhou Goats Offered a 10% Crude Protein Diet Among Four Energy Levels
Source: Animals (Basel). 2025 Jul 23;15(15):2174. doi: 10.3390/ani15152174 (PMC12345419; doi:10.3390/ani15152174)
Supplement: Supplementary file 1 [file animals-15-02174-s001.zip › animals-3723803-supplementary.pdf]

Table S1 Composition of dietary ingredients and nutritional levels of the 4 treatments.

Items	LE	MLE	MHE	HE
Ingredient, % of DM				
Oat hull	50.0	47.0	43.0	29.0
Rice hull meal	30.0	18.0	7.00	6.00
Corn, ground	1.70	7.10	14.05	23.95
Soybean meal	10.5	7.60	4.00	2.50
Wheat bran	1.60	8.00	16.00	22.00
Soybean hulls	2.00	5.90	7.45	4.10
Palm meal	0.10	1.00	3.10	5.65
Soybean oil	0.10	1.40	1.40	2.80
Commercial Premix[1]	4.00	4.00	4.00	4.00
Nutrient composition, % of DM				
Dry matter	93.2	94.6	96.8	93.4
Crude protein	9.91	9.88	9.90	9.93
Ether extract	3.88	5.03	5.33	6.72
Neutral detergent fiber	71.1	66.2	57.6	48.0
Acid detergent fiber	30.1	25.5	22.0	19.6
Metabolizable energy	7.01	8.33	9.66	10.98

LE = low energy group, 7.01 MJ/kg DM; **MLE** = middle low energy group, 8.33 MJ/kg DM; **MHE** = middle high energy group, 9.66 MJ/kg DM; **HE** =high energy group, 10.98 MJ/kg DM; **SEM** = standard error of the means.

[1] Premix (per kg) contains: Cu 250 mg, Fe 1,250 mg, Zn 1,000 mg, Mn 1050 mg, I 30 mg, Se 30 mg, Co 50 mg, Vitamin A 100 kIU, Vitamin D3 4,500 IU, Vitamin E 900 mg, Vitamin K3 45 mg.
